# Nutrient intakes and sources of fiber among children with low and high dietary fiber intake: the 2016 feeding infants and toddlers study (FITS), a cross-sectional survey

**DOI:** 10.1186/s12887-019-1822-y

**Published:** 2019-11-18

**Authors:** Kristen Finn, Emma Jacquier, Brian Kineman, Heidi Storm, Ryan Carvalho

**Affiliations:** 1 Nestlé Nutrition, 1812 N Moore St, Arlington, VA 22209 USA; 2 Nestlé Research, Route du Jorat 57, 1000 Lausanne, Switzerland; 3 Nestlé Nutrition, Rue d’Entre-deux-Villes 10, 1814 La Tour-de-Peilz, Switzerland

**Keywords:** Pediatrics, Fiber, Macronutrients, Micronutrients

## Abstract

**Background:**

Increasing dietary fiber intake in children may improve overall diet quality. The purpose of this study was to compare nutrient intakes and sources of fiber between young children with low and high fiber intakes utilizing data from the Feeding Infants and Toddlers Study (FITS) 2016.

**Methods:**

The FITS 2016 was a nationwide, cross sectional survey of caregivers designed to assess food and nutrient intakes, feeding behaviors, and dietary patterns among infants and young children living in the U.S. Energy adjusted macro and micronutrient intakes (nutrients/1000 kcals) of children with energy adjusted fiber intakes (g/1000 kcals) in the highest quartile were compared to those in the lowest quartile with paired t-tests. Sources of fiber for each quartile were ranked according to percent of total fiber intake.

**Results:**

Children with fiber intakes in the highest quartile had significantly lower intakes of total fat (mean difference ranged from 7.4–9.6 g, *p* < 0.0005) and saturated fat (mean difference ranged from 4 to 5.8 g, *p* < 0.0005), and significantly higher intakes of vitamin B-6 (mean difference ranged from 0.3–0.4 mg, *p* < 0.0005), magnesium (mean difference ranged from 57.2–61.8 mg, *p* < 0.0005), iron (mean difference ranged from 2.2–3.7 mg, *p* < 0.0005), and potassium (mean difference ranged from 318.2 mg to 446.1 mg, *p* < 0.0005) compared to children in the lowest quartile across all age groups. Children in the highest quartile had higher intakes of nut butters, legumes, fruits, and vegetables and consumed a greater percentage of grains as whole grains than those in the lowest quartile.

**Conclusion:**

Encouraging intake of fruits, vegetables, legumes, nut butters, and at least 75% of grains as whole grains may help young children improve dietary fiber intake and overall diet quality.

## Background

Dietary fiber has many physiological effects including reducing postprandial glucose concentrations, improving fecal bulk, promoting laxation, interfering with fat and cholesterol absorption, and altering bacteria populations in the gut microbiome [1–6]. The benefits of dietary fiber intake in adults have been well documented, and include improved body mass index, reduced cardiovascular disease risk, reduced type two diabetes risk, and reduced risk of colorectal and breast cancers [7–12]. The direct health benefits of dietary fiber intake in children are less established, but the role of dietary fiber intake during childhood in lowering the risk of developing constipation, obesity, diabetes, and heart disease has been explored [5, 7, 13]. Higher intake of whole grains, a source of dietary fiber, has been associated with improved overall diet quality among children and adults [14–16].

Dietary fiber recommendations for children vary worldwide and have increased over the past two decades in the United States (U.S.) [5, 17–20]. In Europe, the European Food Safety Authority (EFSA) set the dietary reference value for dietary fiber for children over 1 year of age at 2 g per MJ per day (~ 8.4 g per 1000 kcal or ~ 11.4 g for children aged 1 to 3 years) based on the amount of dietary fiber that is adequate for normal laxation [17]. Historically in the U.S., it was recommended that foods rich in fiber be introduced gradually during complementary feeding to about 5 grams per day by the end of the first year [20]. For children over 2 years, dietary fiber recommendations were based on the American Health Foundation’s “age plus 5 rule” which translates to a level of 6 g per day for 1 year old children, 7 g per day for 2 year old children, and 8 g per day for 3 year old children [20, 21]. In 2002, the Institute of Medicine (IOM) established an adequate intake (AI) level for fiber for children [5]. Although complementary food, which may include dietary fiber, is introduced around 6 months of age in the U.S., there was no available data on fiber intake in this age group at the time the most recent dietary reference intakes (DRIs) for dietary fiber were established, so no AI level was set [5]. The current AI level for children aged 1–19 years was based on adult data demonstrating that 14 g/1000 kcal reduces the risk of coronary heart disease [5]. The AI was set by extrapolating the adult ratio for the median energy intake for each age and gender group of children and translates to a level of 19 g per day for children aged 1 to 3 years, but the safety and feasibility of this recommended level has been questioned [5, 13, 20].

Many children are falling short of the IOM recommended guidelines for fiber intake. The 2016 Feeding Infants and Toddlers Study (FITS) revealed that only 3.2, 9.1, and 7.5% of 12–23.9, 24–35.9, and 36–47.9 month old children respectively were meeting the AI for dietary fiber [22]. The mean intake was 10 ± 0.1 g per day for children aged 12–23.9 months and 12 ± 0.3 g per day for children aged 24–47.9 months [22]. This is consistent with other studies of young children in the U.S. which show that the average intake of fiber was 7-9 g per day among toddler aged children (1–2 years) and 10–11.5 g per day among preschool aged children (3–5 years) [14, 23–26]. Since very few children met the AI for dietary fiber, a more modest goal may be more attainable and still be associated with increased levels of other beneficial nutrients.

There are several factors that may contribute to the under consumption of fiber rich foods among young children in the U.S. Whole grain intake has been positively correlated with family income [27]. Vegetable consumption in children has been linked to parental consumption, frequency of vegetable offerings, and the sensory characteristics of the vegetable [28]. Infrequent fruit and vegetable consumption during complementary feeding is associated with the same behavior at 6 years of age [29]. Targeting low income families and encouraging frequent offerings of fruit, vegetables, and whole grains during complementary feeding and early childhood may be key to increasing fiber consumption among children.

We hypothesized that children with high dietary fiber intakes (those in the highest quartile) would have diets higher in beneficial micronutrients such as iron, vitamin C, vitamin D, and B vitamins and lower intakes of saturated fat compared to children with low dietary fiber intakes (those in the lowest quartile). The aims of this study were to determine (1) the energy adjusted (EA) dietary fiber intakes (including soluble and insoluble fiber) of children aged 12–47.9 months, (2) the characteristics of children aged 12–47.9 months with low dietary fiber intake (EA dietary fiber intakes in the lowest quartile) and those with high dietary fiber intake (EA dietary fiber intakes in the highest quartile), (3) the differences in EA intakes of macronutrients and micronutrients between children with low and high dietary fiber intake, (4) the sources of dietary fiber (including soluble and insoluble fiber) among children with low and high dietary fiber intakes, and (5) the contribution of major food groups to total food and beverage intake among children with low and high dietary fiber intake. The findings of this study may provide evidence that young children with fiber intakes at the high end of the normal distribution of fiber intake also have higher intakes of other beneficial nutrients. Discerning important sources of dietary fiber may help guide recommendations for improving dietary fiber intake in young children.

## Methods

The FITS 2016 was a nationwide, cross sectional survey of caregivers designed to assess food and nutrient intakes, feeding behaviors, and dietary patterns among infants and young children living in the U.S. [30]. FITS 2016 was a follow up to two previous surveys, FITS 2002 and FITS 2008. Eligibility criteria to participate included the presence of a child less than 4 years of age in the household, the presence of an adult primary caregiver knowledgeable about the child’s diet, and a willingness to participate. Compared to the U.S. population of adults in households with a child younger than 4 years of age, the survey respondents were less likely to be Hispanic, more likely to be white, and had higher education levels. To control for bias between the survey estimate and the true U.S. population, the sample was weighted first according to the probability of being selected, unknown eligibility, nonresponse, and number of eligible children in the household and then according to U.S. population totals for race/ethnicity of the child by age category, sex of child by age category, age category of child by WIC status, census division, and education level of the caregiver. Further details regarding sample weights are provided in a previous publication focused on FITS methodology [30]. Caregivers of children aged 0–47.9 months (*n* = 3235) completed a 24 h dietary recall (24 HDR) via a telephone interview conducted by trained dietary interviewers at the University of Minnesota. A second 24 HDR was completed by a random subset of the sample to estimate usual nutrient intake distributions (*n* = 799). The Nutrition Data System for Research (NDSR, version 2015: University of Minnesota, Minneapolis, MN) was used to collect and analyze the dietary intake data. The NDSR relies on the Nutrition Coordinating Center (NCC) Food and Nutrient Database [31]. Food groups and subgroups were defined for this project and align closely with the food grouping system used in the National Health and Nutrition Examination Survey. Small range age groups were selected to capture changes in feeding practices that occur in early childhood. All study methods and procedures were approved by the RTI International, University of Minnesota, Docking Institute of Public Affairs, and Fort State University Institutional Review Boards. All participants provided informed consent. The FITS 2016 study design and methods including sampling and weighting methodology have been described in detail elsewhere [30].

For this analysis, the first 24 HDR for children 12–47.9 months old was used (*n* = 1733). Infants were omitted since there is no AI level established for this age group and there are no current recommendations for dietary fiber intake from complementary foods only. Dietary fiber was defined as all non-digestible carbohydrates including cellulose, gums, hemi-cellulose, lignin, muscilages, and pectins according to the NCC database. Since children with higher fiber intakes may also have higher overall energy and nutrient intakes, the mean and quartiles for EA dietary fiber intakes (g/1000kcals) were calculated for all children aged 12–23.9 months, 24–35.9 months, and 36–47.9 months. Characteristics including race, household income, caregiver education, and participation in the Women, Infants, and Children (WIC) supplemental nutrition program of children in the lowest and highest quartiles of EA dietary fiber intake were extracted. The EA nutrient intakes (macronutrients and micronutrients) of children in the highest and lowest quartile of EA dietary fiber intake were calculated for each age group. The food sources of dietary fiber were ranked according to percentage contribution to total dietary fiber intake for those in the lowest and highest quartiles for EA fiber intake for each age group. All sources that contributed at least 2% of the total dietary fiber intake were reported. The percentage of intake from the major food groups was calculated based on total grams of food and beverage intake and grams of intake from each food group (dairy, fruits, vegetables, grains, proteins (meat and non-meat), sweets and sweetened beverages, savory snacks, mixed dishes, sauces and condiments, fats and oils, water).

Differences in characteristics of those in the lowest and highest quartiles of EA dietary fiber intake for each age group were analyzed using paired t-tests. Mean intakes of EA macronutrients and micronutrients were calculated and analyzed for statistically significant differences between those in the lowest compared to those in the highest quartile of EA dietary fiber intake for each age group using paired t-tests. A Bonferroni-corrected *p* value of 0.002 was used to determine statistical significance. Descriptive statistics were calculated for food group intakes. SAS (version 9, SAS Institute Inc.: Cary, NC) and SAS-callable SUDAAN® (version 11, RTI International: Research Triangle Park, NC) software was used for all statistical analyses.

## Results

The mean EA dietary fiber intakes were 8.6 ± 0.16 g, 9.0 ± 0.28 g, and 8.4 ± 0.23 g per 1000 kcals among children aged 12–23.9, 24–35.9, and 36–47.9 month olds respectively. The 25th percentile for EA dietary fiber intake was 5.63 g, 6.26 g, and 5.89 g per 1000 kcals among 12–23.9, 24–35.9, and 36–47.9 month olds respectively. The 75th percentile for EA dietary fiber intake was 10.7 g, 10.9 g, and 10.2 g per 1000 kcals among 12–23.9, 24–35.9, and 36–47.9 month olds respectively.

Some characteristics of children in the lowest and highest quartile of fiber intake differed (See Table 1). A higher percentage of mothers had a college degree among children in the highest quartile compared to the lowest quartile of EA dietary fiber among 12–23.9 month and 36–47.9 month old children. A higher percentage of children were participating in the WIC program in the lowest quartile than the highest quartile among children aged 12–23.9 months. A higher percentage of children were in households with low annual earnings in the lowest quartile compared to the highest quartile among children aged 12–23.9 months.

**Table 1:** Characteristics of Children in Lowest and Highest Quartile of EA Dietary Fiber Intake (Weighted Estimate (Standard Error of Weighted Estimate)^a^)

	12–23.9 Months		24–35.9 Months		36–47.9 Months	
Characteristic	≤25th %ile *n* = 259	≥75th %ile *n* = 289	≤25th %ile *n* = 79	≥75th %ile *n* = 75	≤25th %ile *n* = 72	≥75th %ile *n* = 72
Gender % Male	53.7 (4.2)	51.5 (3.8)	57.0 (7.5)	59.9 (7.0)	53.2 (7.1)	56.0 (7.4)
Race						
White	72.3 (4.1)	65.7 (3.9)	62.2 (8.8)	76.1 (6.3)	72.5 (6.6)	65.1 (7.3)
African American	21.3 (3.3)	14.3 (2.4)	22.5 (5.4)	10.9 (3.5)	19.7 (5.4)	18.5 (5.1)
American Indian	1.7 (0.9)	3.6 (1.7)	0.0 (0.0)	1.0 (1.0)	8.8 (4.9)	0.0 (0.0)
Asian	2.4 (1.1)*	9.9 (2.6)*	9.9 (7.0)	3.8 (3.7)	0.0 (0.0)	6.1 (4.2)
Other	13.3 (3.7)	14.1 (3.2)	14.2 (8.7)	16.2 (6.2)	13.2 (5.5)	14.5 (6.3)
Mother’s Education						
High School or Less	47.9 (4.9)*	19.2 (3.7)*	28.9 (8.1)	26.4 (9.0)	56.4 (7.7)	36.8 (8.1)
Some post-secondary	26.8 (3.8)	21.2 (3.6)	17.9 (5.6)	8.3 (3.3)	17.8 (6.0)	10.6 (4.1)
College or above	25.3 (3.4)*	59.7 (4.3)*	53.2 (9.2)	65.3 (8.9)	25.8 (5.9)*	52.5 (8.1)*
Receives WIC % Yes	63.2 (3.6)*	36.7 (3.9)*	38.9 (7.9)	21.6 (6.1)	35.1 (7.1)	33.1 (8.1)
Household Income						
Under 10,000	23.2 (4.5)*	8.2 (2.1)*	12.9 (4.5)	9.7 (6.3)	19.1 (6.4)	5.2 (3.6)
10,000 to 19,999	12.5 (3.3)	7.5 (2.6)	19.3 (8.3)	8.1 (4.9)	10.6 (3.8)	22.8 (6.4)
20,000 to 34,999	21.0 (3.4)*	11.3 (2.3)*	13.0 (4.5)	10.0 (3.7)	19.2 (6.4)	14.1 (8.4)
35,000 to 49,999	14.9 (2.6)	21.7 (3.4)	12.8 (5.2)	7.9 (2.9)	10.3 (5.2)	6.3 (3.6)
50,000 to 74,999	14.6 (2.3)	18.3 (2.7)	7.5 (2.9)	20.8 (5.4)	14.2 (4.5)	12.3 (3.8)
75,000 to 99,999	4.8 (1.1)*	15.2 (2.4)*	5.9 (3.2)*	23.5 (6.2)*	15.9 (5.5)	16.8 (5.5)
100,000 to 149,999	7.1 (2.2)	11.2 (2.0)	23.0 (9.8)	16.2 (5.4)	7.1 (3.8)	13.3 (4.9)
150,000 and over	1.9 (0.8)	6.6 (1.9)	5.7 (3.5)	3.9 (2.1)	3.6 (2.6)	9.3 (4.0)

*WIC* Women, Infants, and Children

^a^Weighted estimates have been provided to control for bias and provide a truer estimate of the US population

*p < 0.002 (Bonferroni correction for multiple t-tests)

Intakes of total fat and saturated fat were significantly lower among children in the highest quartile compared to the lowest quartile across all age groups (See Table 2). Intakes of vitamin B-6, magnesium, iron, and potassium were significantly higher among children in the highest quartile compared to the lowest quartile across all age groups. Intakes of vitamin B-12, calcium, and vitamin D were lower in the highest quartile compared to the lowest quartile among 12–23.9 month old children. The mean intakes of dietary fiber and vitamin D were below the AI for children in both groups across all age groups. The mean intake of vitamin E was below the EAR for children in the lowest quartile of EA dietary fiber intake only among children aged 12–23.9 and 24–35.9 months and in both groups among children aged 36–47.9 months.

**Table 2:** EA Nutrient Intakes (Mean (SE) Nutrient per 1000 kcal)

Nutrient		12–23.9 Months ≤25th %ile *n* = 259 ≥75th %ile *n* = 289	24–35.9 Months ≤25th %ile *n* = 79 ≥75th %ile *n* = 75	36–47.9 Months ≤25th %ile *n* = 72 ≥75th %ile *n* = 72
Total Fat (g)	DRI	Not Determened	Not Determened	Not Determened
	≤25th %ile	42.5 (0.6)*	39.8 (0.8)*	39.1 (1.1)*
	≥75th %ile	32.9 (0.6)*	30.5 (1.4)*	31.7 (1.6)*
Saturated Fat (g)	DRI	Not Determened	Not Determened	Not Determened
	≤25th %ile	18.0 (0.4)*	14.6 (0.4)*	14.3 (0.6)*
	≥75th %ile	12.2 (0.3)*	10.6 (0.6)*	10.2 (0.7)*
Vitamin E (mg)	DRI	EAR = 5 mg	EAR = 5 mg	EAR = 5 mg
	≤25th %ile	3.1 (0.1)*	3.4 (0.2)**	3.4 (0.2)
	≥75th %ile	5.1 (0.3)*	5.1 (0.5)**	4.3 (0.3)
Vitamin C (mg)	DRI	EAR = 13 mg	EAR = 13 mg	EAR = 13 mg
	≤25th %ile	47.3 (4.4)*	42.7 (5.3)*	33.5 (3.8)
	≥75th %ile	70.4 (3.5)*	72.7 (5.7)*	54.8 (6.8)
Vitamin A (mcg RAE)	DRI	EAR = 210mcg	EAR = 210mcg	EAR = 210mc
	≤25th %ile	460.8 (14.7)*	401.9 (19.4)*	355.5 (25.3)
	≥75th %ile	588.1 (24.8)*	542.1 (32.9)*	410.3 (46.0)
Riboflavin (mcg)	DRI	EAR = 0.4 mg	EAR = 0.4 mg	EAR = 0.4 mg
	≤25th %ile	1.5 (0.0)*	1.3 (0.0)	1.2 (0.0)
	≥75th %ile	1.3 (0.0)*	1.3 (0.1)	1.1 (0.1)
Thiamin (mg)	DRI	EAR = 0.4 mg	EAR = 0.4 mg	EAR = 0.4 mg
	≤25th %ile	0.8 (0.0)*	0.8 (0.0)	0.9 (0.0)
	≥75th %ile	1.0 (0.0)*	0.9 (0.0)	0.9 (0.0)
Niacin (mg)	DRI	EAR = 5 mg	EAR = 5 mg	EAR = 5 mg
	≤25th %ile	8.7 (0.3)*	8.2 (0.4)*	9.9 (0.4)
	≥75th %ile	11.1 (0.3)*	11.2 (0.7)*	11.3 (0.4)
Vitamin B-6 (mg)	DRI	EAR = 0.4 mg	EAR = 0.4 mg	EAR = 0.4 mg
	≤25th %ile	0.8 (0.0)*	0.8 (0.0)*	0.8 (0.0)*
	≥75th %ile	1.2 (0.0)*	1.2 (0.1)*	1.1 (0.1)*
Folate Equivalents (mcg)	DRI	EAR = 120mcg	EAR = 120mcg	EAR = 120mcg
	≤25th %ile	208.5 (6.8)*	206.9 (13.0)*	249.3 (18.6)
	≥75th %ile	304.4 (9.6)*	301.4 (22.7)*	305.8 (18.6)
Vitamin B-12 (mcg)	DRI	EAR = 0.7mcg	EAR = 0.7mcg	EAR = 0.7mcg
	≤25th %ile	3.6 (0.1)*	3.2 (0.2)	2.8 (0.1)
	≥75th %ile	2.8 (0.1)*	2.8 (0.2)	2.4 (0.3)
Calcium (mg)	DRI	EAR = 500 mg	EAR = 500 mg	EAR = 500 mg
	≤25th %ile	844.5 (26.0)*	701.0 (32.3)	647.3 (43.9)
	≥75th %ile	730.5 (17.4)*	716.3 (39.8)	547.3 (38.0)
Phosphorus (mg)	DRI	EAR = 380 mg	EAR = 380 mg	EAR = 380 mg
	≤25th %ile	784.3 (15.6)	691.9 (20.3)	673.5 (27.0)
	≥75th %ile	746.0 (11.2)	778.2 (30.9)	693.8 (28.3)
Vitamin D (mcg)	DRI	EAR = 10mcg	EAR = 10mcg	EAR = 10mcg
	≤25th %ile	7.7 (0.3)*	5.2 (0.5)	4.5 (0.4)
	≥75th %ile	4.9 (0.2)*	4.3 (0.4)	3.1 (0.4)
Magnesium (mg)	DRI	EAR = 65 mg	EAR = 65 mg	EAR = 65 mg
	≤25th %ile	122.7 (2.0)*	117.7 (3.8)*	108.2 (4.4)*
	≥75th %ile	179.9 (2.5)*	179.5 (5.3)*	169.8 (9.4)*
Iron (mg)	DRI	EAR = 3 mg	EAR = 3 mg	EAR = 3 mg
	≤25th %ile	5.6 (0.2)*	5.6 (0.4)*	6.1 (0.2)*
	≥75th %ile	9.3 (0.3)*	7.7 (0.4)*	8.3 (0.4)*
Potassium (mg)	DRI	AI = 3000 mg	AI = 3000 mg	AI = 3000 mg
	≤25th %ile	1375.3 (28.9)*	1274.9 (83.6)*	1082.8 (47.1)*
	≥75th %ile	1693.6 (27.6)*	1721.0 (72.7)*	1424.2 (52.7)*
Zinc (mg)	DRI	EAR = 2.5 mg	EAR = 2.5 mg	EAR = 2.5 mg
	≤25th %ile	5.4 (0.1)	4.9 (0.2)	5.1 (0.2)
	≥75th %ile	5.9 (0.1)	5.9 (0.4)	5.7 (0.4)
Sodium (mg)	DRI	AI = 1000 mg	AI = 1000 mg	AI = 1000 mg
	≤25th %ile	1266.5 (30.1)	1541.7 (79.8)	1556.4 (50.5)
	≥75th %ile	1175.5 (31.2)	1399.9 (99.6)	1391.5 (54.3)

*DRI* Dietary Reference Intake, *EAR* Estimated Average Requirement, *RAE* Retinol Activity Equivalents, *AI* Adequate Intake, * = *p* < 0.0005, ** = *p* < 0.002 (Bonferroni correction for multiple t-tests)

The top 2 sources of dietary fiber were grains and fruit (12–23.9 months), and grains and mixed dishes (24–35.9 and 36–47.9 months) among children in the lowest quartile of EA dietary fiber intake (See Table 3). The top 2 source of dietary fiber were fruit and grains in all 3 age groups among children in the highest quartile. Children in the lowest quartile consumed 26–54% of grains as whole grains compared to 74–80% in the highest quartile. Children in the highest quartile consumed more legumes, nuts, and seeds and a greater variety of fruits and vegetables compared to children in the lowest quartile. Of note, nuts and seeds were categorized together and consumed mostly as peanut butter.

**Table 3:** Ranked Sources of Dietary Fiber (includes all sources ≥2% of total fiber intake)

	12–23.9 Months				24–35.6 Months				36–47.9 Months			
	≤25th %ile *n* = 259		≥75th %ile *n* = 289		≤25th %ile *n* = 79		≥75th %ile *n* = 75		≤25th %ile *n* = 72		≥75th %ile *n* = 72	
Rank	Food	%	Food	%	Food	%	Food	%	Food	%	Food	%
1	Grains	26	Fruit/Juice	31	Mixed Dishes	23	Fruit/Juice	32	Grains	27	Grains	27
	Whole grains	14	-Fruits	26	-Pizza	5	-Fruit	29	Whole Grains	7	Whole Grains	21
	-Cereal^a^	9	Banana	8	-Sandwich	4	Apple	8	-Bread^a^	10	-Bread^a^	7
	Sweetened^b^	5	Apples	4	Peanut butter	3	Banana	8	-Cereal^a^	7	Whole grain	6
	Whole grain^b^	8	Berries	4	-Beef dishes	3	Orange	4	Whole grain^b^	5	-Cereal^a^	9
	-Bread^a^	6	Avocado	3	-Macaroni & cheese	3	Berries	3	Presweetened^b^	4	Non-sweet^b^	5
	-Crackers^a^	3	Citrus Fruits	2	-Pasta dishes	2	Pear	2			Whole grain^b^	8
	-Infant Cereal^a^	2	Orange	2	-Burrito, taco, nachos	2					-Crackers	5
	-Pancakes/Waffles^a^ -Pasta/Rice^a^	2 2	Other -Baby foods	6 3	-Soup	2					-Pasta/rice -Pancakes/Waffles	3 2
2	Fruit/Juice	20	Grains	25	Grains	20	Grains	19	Mixed Dishes	18	Fruit/Juice	25
	-Fruits	13	Whole Grains	20	Whole Grains	8	Whole Grains	14	-Pizza	6	-Fruits	24
	Apple	5	-Cereal^a^	9	-Bread^a^	5	-Cereal^a^	8	-Macaroni & cheese	4	Apple	6
	Bananas	5	Non-sweet^b^	6	-Cereal^a^	6	Non-sweet^b^	6	-Pasta dishes	4	Banana	8
	−100% Juice	5	Whole grain^b^	9	Sweetened^b^	4	Whole grain^b^	7	-Sandwiches	3	Other	4
			-Bread^a^	6	Wholegrain^b^	4	-Bread^a^	5			Berries	2
			-Pasta/rice^a^	4	-Pancakes/waffles^a^	4	-Pasta/rice	3			Orange	2
			-Crackers^a^	2	Pancakes	3	-Pancakes/waffles^a^	2			Pear	2
			-Pancakes/waffles^a^	2	-Pasta/rice	2	Pancakes	2			-Juice	2
			-Infant cereal^a^	2								
3	Mixed Dishes	19	Vegetables	16	Fruit/Juice	18	Meat/Protein	14	Fruit/Juice	14	Meat/Protein	16
	-Pasta dishes	4	-Other	4	-Fruits	14	-Non-Meat protein Legumes Peanut butter/nuts/seeds	14 8 5	-Fruit	11	-Non-meat protein Legumes Peanut butter/nuts/seeds	15 10 5
	-Macaroni & cheese	3	Green beans	2	Bananas	4			Banana	4		
	-Pizza	3	-Dark green	3	Apple	3			Apple	2		
	-Soup	2	Broccoli	2	Melon	2			Grapes	2		
	-Sandwich	2	-Orange/red	3	-Juice	4			-Juice	2		
	-Baby food dinners	2	-Starchy	2								
			-White potato	2								
			-Baby food	2								
4	Vegetables	9	Meat/Protein	11	Sweets	14	Vegetables	13	Sweets	13	Vegetables	11
	-White potato	7	-Non-meat Protein	10	-Sweet bakery	5	-Orange/red	4	-Cereal Bars	7	-Other	4
	Fried	3	Legumes	8	Cookies /brownies	3	Carrots	2	-Sweet bakery	6	-Orange/red	2
	Mashed	3	Peanut butter, nuts, seeds	2	-Candy	2	-Other	4	Cookies/brownies	3	-White potato	2
	-Starchy	3			-Sweetened beverages	2	-Dark green	2	-Sweetened beverages	2		
	Corn	2					Broccoli	2				
	-Other	3										
	Green beans	2										
	-Orange/red	2										
5	Sweets	9	Mixed Dishes	8	Vegetables	12	Mixed Dishes	12	Vegetables	12	Mixed Dishes	10
	-Sweet bakery	4	-Pasta dishes	2	-Orange/red	2	-Bean dishes	4	-Other	3	-Sandwiches	3
	Cookies/brownies	3			-Other	2	-Soup	3	Green beans	2	-Bean dishes	2
					Green beans	2			-White potato	7	-Pasta dishes	2
					-White Potato	7			Fried	4		
					Fried	6			Mashed	2		
6	Meat/Protein	6	Sweets	5	Savory Snacks	6	Sweets	6	Savory Snacks	7	Sweets	5
	-Non-meat protein	3	-Sweet bakery	2	-Corn chips	3	-Cereal bars	3	-Chips	4	-Cereal bars	3
					-Chips	2			-Puffs	2	-Sweet bakery	2
7	Savory Snacks	2	Dairy	3	Meat/ Protein	4	Dairy	3	Meat/Protein	5	Savory snacks	2
									Peanut butter/nuts/seeds	2	-Chips	2
8	Dairy	2	Savory Snacks	2	Dairy	2			Dairy	3	Dairy	2
			-Chips	2								

^a^Whole grain and non-whole grain combined, ^b^All cereals were categorized as both whole grain/non-whole grain and sweetened/non-sweetened

**Fig. 1:**
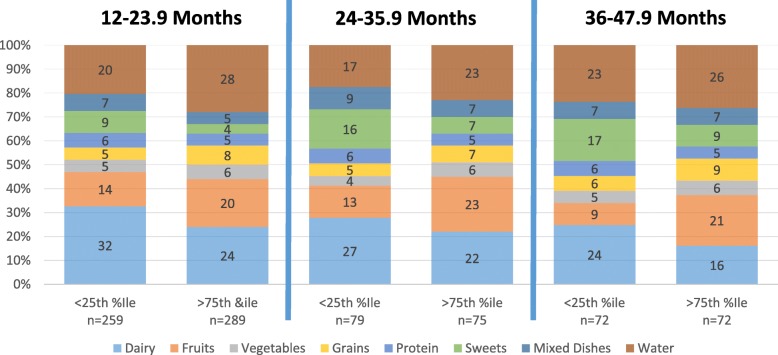
Percentage of Total Food and Beverage Intake from Food Groups*. *May not add up to 100% as ≤1% of intake may have come from savory snacks and/or condiments and sauces

Children in the lowest quartile of energy adjusted dietary fiber intake consumed a higher percentage of total food and beverage intake as dairy products and sweets compared to those in the highest quartile (See Fig. 1). Children in the highest quartile of energy adjusted dietary fiber intake consumed a higher percentage of total food and beverage intake as fruit compared to those in the lowest quartile. Food intake from the fats and oils, savory snacks, and sauces and condiments groups was absent or negligible in the entire sample.

## Discussion

In this study, children with EA dietary fiber intakes (including soluble and insoluble fiber) in the highest quartile had significantly lower intakes of total fat and saturated fat and significantly higher intakes of several beneficial nutrients compared to children with dietary fiber intakes in the lowest quartile across all age groups. These findings are consistent with those of Hampl et al. who found that 4 to 10 year old children who met fiber recommendations (age plus 5 rule) had lower intakes of total fat and higher intakes of vitamin A, magnesium, folate, and iron compared to those who did not [16]. Our findings are also consistent with O’Neil et al. who found that intakes of fiber, folate, magnesium, vitamin B6, and vitamin A were higher while intakes of total fat and saturated fat were lower among 2 to 5 year old children who consumed at least 3 servings of whole grains per day compared to those who did not [14]. Of interest, we found that among toddlers aged 12–23.9 months old, intakes of calcium and vitamin D were significantly lower among those in the highest quartile of EA dietary fiber intake compared to those in the lowest quartile; however, both groups had intakes below the recommended level for vitamin D. This is likely due to differences in milk intake.

Hampl et al. found that children who met fiber recommendations (age plus 5 rule) had higher intakes of cereals and breads, nut butters, legumes, fruits and vegetables than those who did not meet the recommendations [16]. This is consistent with our findings that children in the highest quartile of EA dietary fiber intake had higher intakes of nut butters and legumes, and consumed a greater volume and variety of fruits and vegetables compared to those in the lowest quartile. We also found that children in the highest quartile of EA dietary fiber intake consumed a greater percentage of their grains as whole grains compared to children in the lowest quartile (74–80% vs. 26–54%), but this needs to be confirmed in other studies.

Children in the lowest quartile of EA dietary fiber consumed a greater percentage of total food and beverage intake from sweets and lower percentage as fruits compared to those in the highest quartile. This is consistent with findings from Fox et al. who found that desserts such as cookies, cakes, and pies were a top contributor to total grain intake among 2 and 3 year olds in the U.S. [32]. Encouraging intakes of fruit in lieu of sweets may be one strategy to improve dietary fiber intakes in this population.

Recently, the American Academy of Pediatrics (AAP) published a policy statement advocating for a focus on key nutrients for brain development including protein, long-chain polyunsaturated fatty acids, choline, folate, iodine, iron, zinc, and vitamins A, D, B6, and B12 [33]. In our study, higher intakes of dietary fiber were associated with increased intakes of some of these key nutrients including folate, iron, vitamins A and B6. The AAP also recommends that beneficial dietary choices in healthy eating be advocated rather than focusing only on the avoidance of unhealthy foods [33]. Focusing on adequate dietary fiber intake at initiation of complementary feeding may be a strategy to help parents incorporate healthy foods into their child’s diet and lead to higher intakes of foods like fruits, vegetables, whole grains, nuts, seeds, and legumes which are naturally rich in fiber and other key nutrients.

In our sample, caregivers of children in the low fiber quartile were less educated and had lower household incomes compared to those in the high fiber quartile, especially among children aged 12–23.9 months. This is consistent with other findings that income impacts the quality of household food purchases [34]. The Women, Infants, and Children program and the Child and Adult Care Food Program are designed to provide healthy foods, including high fiber foods, to low income children. Both programs currently promote fruit, vegetable, and whole grain intake, but also allow for some refined grains which is consistent with current recommendations [35, 36].

The current IOM recommendations for dietary fiber for young children (14 g/1000 kcals, 19 g/day) are based on clinical data from adult studies which is in contrast to the EFSA recommendations which are based on the amount of fiber necessary for normal laxation in children [5, 17]. We found that dietary fiber intakes of at least 10 g/1000kcals, which are slightly higher than the EFSA recommendations, are attainable by a greater percentage of children aged 1 to 3 years compared to the IOM recommendations (~ 25% vs. < 10%) and were associated with improvements in intakes of several key nutrients.

The Dietary Guidelines for Americans 2015–2020 which are designed for Americans aged 2 years and older, recommend that at least half of grains be consumed as whole grains [37]. Shifting from low fiber to high fiber grain based foods was proposed as a feasible strategy to increase fiber intake by a group of leading nutrition and fiber experts who were tasked with probing realistic solutions for closing the fiber gap [38]. We observed that young children with EA dietary fiber intakes in the highest quartile consumed 74–80% of grains as whole grains. A recommendation of consuming a higher percentage of grains as whole grains (75%) may be necessary to reach dietary fiber recommendations in this age group. Consumers often equate ‘whole grain” label claims with fiber intake, but many products with such claims are not a “good source” of fiber (<3 g/serving) [39]. Education should focus on recognizing whole grain foods that are also high in dietary fiber. Prospective dietary intervention studies directed at increasing intake of dietary fiber in young children may be helpful to determine if such a strategy results in improved overall diet quality.

The FITS study is the only nationally representative, cross-sectional survey of infant and toddler feeding practices in the U.S. This is the only analysis to date that specifically explores the associations between EA dietary fiber intake by quartile and nutrient intakes. Findings were presented in small age groups to capture the changes in feeding practices that occur in early life.

There are several limitations to this study. We relied on a 24 HDR to assess dietary intakes and were unable to assess changes in EA dietary fiber and nutrient intakes over time. The sample sizes in the 24–35.6 month and 36–47.9 month age groups were small. Since this was a dietary survey, we were unable to determine if EA dietary fiber intakes were associated with conditions such as constipation, obesity, and diabetes. The nutrient intakes reflect intakes from total diet including dietary supplements. The observed differences in nutrient intakes between the two groups are consistent with the differences in dietary fiber sources; however, we did not assess if more infants in one group were receiving supplements compared to the other. The FITS was not specifically designed to examine fiber intakes and thus, foods were not grouped according to their fiber content. Further, we did not distinguish between dietary fibers naturally occurring in foods and those that were added during processing such as guar gum, pectin, and psyllium and we were unable to estimate how much of the total dietary fiber may have come from added fibers. Finally, while these findings are relevant to young children in the U.S., they cannot be generalized to other populations where food systems and sources of dietary fiber may differ.

## Conclusions

In conclusion, the young children in this nationwide survey fell short on dietary fiber. Very few children in our sample met the current IOM recommendation of 14 g/1000kcals. We found that young children with EA dietary fiber intakes of around 10.5 g/1000kcals per day had improved intakes of several key nutrients. Children with higher intakes of dietary fiber consumed at least 75% of grains as whole grains and consumed greater amounts of fruits, vegetables, nut butters, and legumes. Encouraging intakes of foods naturally high in fiber and fruits in lieu of sweets may be an effective strategy to implement the AAP recommendation to improve key nutrient intakes while focusing on positive dietary choices in young children.

## Data Availability

The datasets used and/or analyzed during the current study are available from the corresponding author on reasonable request. Detailed information regarding the survey tools and data collection methodology is available in a separate publication [30].

## Abbreviations

24 HDR: 24 h dietary recall
AAP: American Academy of Pediatrics
AI: Adequate intake
DRI: Dietary reference intake
EA: Energy adjusted
EFSA: European Food Safety Authority
FITS: Feeding Infants and Toddlers Study
IOM: Institute of Medicine
NDSR: Nutrition Data System for Research
NCC: Nutrition Coordinating Center
US: United States
WIC: Women, Infants, and Children
